# Low-dose brachytherapy for early stage penile cancer: a 20-year single-institution study (73 patients)

**DOI:** 10.1186/s13014-016-0676-9

**Published:** 2016-07-27

**Authors:** A. Cordoba, A. Escande, S. Lopez, L. Mortier, X. Mirabel, B. Coche-Déqueant, E. Lartigau

**Affiliations:** Academic Radiation Oncology Department, Oscar Lambret Comprehensive Cancer Center, SIRIC ONCOLille and University Lille 2, 3 rue Fréderic Combemale, Lille, France

**Keywords:** Penile cancer, Interstitial brachytherapy, Local control

## Abstract

**Purpose/objectives:**

The aim of this study is to analyze the results of exclusive interstitial brachytherapy (IBT) as a conservative approach in the treatment of penile cancer confined to the glans or the shaft with long-term follow-up in a single institution.

**Materials/methods:**

Between July 1992 and November 2013, 73 consecutive patients with non-metastatic invasive penile cancer were treated by Low dose rate (LDR) IBT in our institution. The localization of the primary lesion was glands in 67 patients (91.8 %) and shaft in 6 patients (8.2 %). All 73 patients presented with squamous cell carcinoma with grades of differentiation as follows: 34 patients with grade 1 (44.7 %), 9 patients with grade 2 (11.8 %), 9 patients with grade 3 (11.8 %) and 21 patients unknown (28.8 %). Six patients (7.8 %) presented with in situ carcinoma, 55 patients (75,3 %) presented with T1, 11 patients (15 %) presented with T2, and one patient (1.3 %) presented with Tx. Inguinal nodal dissection was performed in 29 patients (38.2 %); 13 patients (17.8 %) presented with histologically confirmed positive ganglion. After circumcision, IBT was performed using a hypodermic needle. The median dose delivered was 60 Gy (range, 40 to 70 Gy). The median activity of the iridium-192 wire was 1.12 mCi/cm, and the median reference isodose rate was 0.4 Gy/h (range, 0.2–1.2). Patients with histological inguinal metastases received external beam radiotherapy to the selected inguinal affected area with a median dose of 45 Gy (30–55 Gy).

**Results:**

The median follow-up time was 51.8 months (range 34.4 to 68.7). The 5-year overall survival was 82.0 %, with eight deaths from cancer and five non-cancer-related deaths. Disease-specific survival was 91.4 %, relapse-free survival was 64.4 %, and local relapse-free survival as 74 %. Total or partial penile preservation was 87.9 % at 5-years. Complications rates at 5 years were 6.6 % urethral stenosis (five patients), two patients (2.6 %) with pain related to sexual intercourse and four patients (5.3 %) with dysuria grade 2. Five patients (6.8 %) required penile amputation for necrosis.

**Conclusions:**

IBT provides good local control with organ preservation, excellent tolerance and low complication rates in early-stage penile cancers.

## Introduction

Penile cancer is a rare tumor, with an incidence rate of only 0.4 to 0.6 % of all malignancies that affect men in Europe [1, 2]. The incidence rates are higher in developing countries [3]. Phimosis, poor hygiene, lack of circumcision during childhood, human papilloma virus 16 (HPV), and high-risk sexual behavior have been previously established as known risk factors [3–6]. The presence of metastatic disease in the lymph nodes is the most important prognostic factor. Sentinel node biopsy or inguinal lymph node dissection is recommended in stages T1-T3 N0-X [7]. Historically, surgical treatment has been performed on primary lesions with psychological alterations and physical morbidities, such as urinary disorders [8, 9]. Conservative treatment must always be performed during the early stages of disease [9], and surgery is the treatment of choice after local failure. In this sense, interstitial brachytherapy (IBT) is an excellent alternative to radical procedures in early-stage penile carcinoma due to the similar outcome and penile conservation rates of approximately 75 % that it affords [10]. According to international recommendations, both LDR and pulsed dose rate (PDR) modalities are used to deliver this treatment [11]. We present a single-institution experience based on 73 cases of invasive penis cancer treated with interstitial brachytherapy with curative intent as the primary treatment in our center from 11/1992 to 11/2013.

## Materials and methods

### Study population and dataset

We recorded all of the patients treated at our institution with a low dose of IBT with iridium-192 with curative intent for squamous cell penile carcinoma from 11/1992 to 10/2013. Data were collected from paper and/or computer medical records. The types of data collected from each patient and tumor are reported in Table 1.

**Table 1:** Patients and tumor characteristics

	Nb	Percent
Patients	73	100
Age (years)		
Mean	60,46	
Tobacco		
Yes	22	30.1 %
No	51	69.9 %
Vascular disease		
Yes	27	36.9 %
No	46	63.0 %
Diabetes		
Yes	10	13.9 %
No	63	78.8 %
Conjunctival tissue disease		
Yes	3	4.1 %
No	70	95.9 %
Anatomopathological tumor grade		
x	21	28.8 %
1	34	46.6 %
2	9	12.3 %
3	9	12.3 %
Localisation		
Glans	67	91.8 %
Shaft	6	8.2 %
Necrosis		
Yes	69	94.5 %
No	4	5.5 %
T		
In situ	6	8.2 %
T1	55	75.3 %
T2	11	15 %
Tx	1	1.3 %
N		
N+	13	17.8 %
N-	60	82.2 %

### Brachytherapy technique treatment

**Fig. 1:**
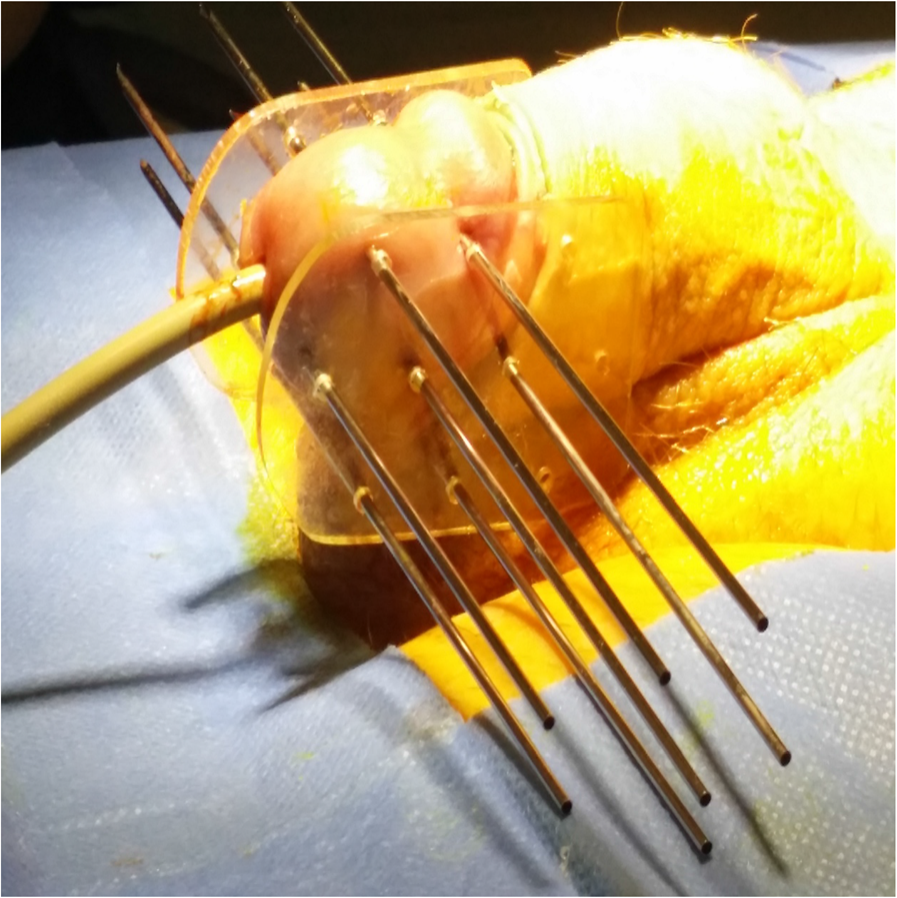
Brachytherapy technique treatment

Under general anesthesia, a radiation oncologist conducts a clinical exam to define the clinical target volume (CTV) that comprises primary tumor with 1 cm of margin in all directions and decides the number of plans and the necessary needle to cover it. Two perforated plates are positioned on both sides of the gland. Each plate has equidistant holes ranging between 1 and 1.5 cm; the choice of a plate type varies depending on the size of the lesion and the judgment of the radiation oncologist. The number of active lines with iridium-192 used for the treatment of the patient is determined by the radiation oncologist. Considering that contouring and planning system had no tools to delineate tumor or organs at risk, implant tried to cover primary tumor with margin leaving if possible a free zone glans and urethra without irradiation. The catheters are fixed to both sides of the plate (Fig. 1). Paris system dosimetry was used to perform dose calculation.

### Statistical analysis

All patients were included in the analysis. The Kaplan-Meier method was performed to estimate the overall survival (OS), overall specific survival (OSS), progression-free survival (PFS), local disease-free survival (LFS), regional disease-free survival (RFS), loco-regional disease-free survival (LRFS) and amputation-free survival (AFS). A log-rank test was performed for all univariate comparative survival. Multi-variable Cox regression for all survival comparison were performed with all clinically interesting variables (considered such if *p* < 0.2). *P*-values are two-tailed and are considered statistically significant if < 0.05. SPSS (IBM Corp. Released 2012. IBM SPSS Statistics for Windows, Version 21.0. Armonk, NY: IBM Corp.) was used for statistical analysis. After treatment, local care and anesthetics were used to relieve local symptomatology.

## Results

Seventy-three patients were treated at Oscar Lambret Cancer Center from 11/1992 to 11/2013.

### Penile radiation treatment

The IBT median dose was 60 Gy (40–70), with more than 75 % of patients receiving a dose at least equal to 60 Gy. The median wire number was 6 (2–17), the median plan number was 3 (1–4), the median wire length was 4 cm (2–6), and the median activity was 1.11 mCi/cm (0.590–1.710). IBT treatment characteristics is reported in Table 2. After treatment, patients received local care consisting in

**Table 2:** IBT treatment characteristics

Interstitial brachytherapy treatment	
Median IBT dose	60 Gy (40 to 70Gy)
Isodose rate	0.2 to 1.2Gy/h
Median BT reference	0.4Gy/h

### Node treatment

Twenty-eight (38.5 %) patients had node surgery, 10 (13.7 %) had bilateral lymphadenectomy, and 18 (24.7 %) had sentinel node surgery. Fourteen patients (19.17 %) presented with one or more positive nodes after inguinal examination and received external radiation therapy to the selected inguinal-pelvic affected area with median dose of 45 Gy (30–55Gy).

### Toxicity (CTCAE-NCI 4.0 score)

Fifteen patients (20.5 %) presented with late toxicity: nine patients (12.3 %) had late dermatitis (two with grade 1, six with grade 2 and one with grade 3), four patients (5.5 %) presented with late urinary trouble, five patients (6.8 %) presented with late stenosis, and two patients (2.1 %) presented with sexual pain.

### Survival rates

The median follow-up time was 51.8 months (range 1.4–156.4). Nine patients (12.3 %) died during the follow-up period, but only three died from cancer (4.1 %), one died from renal failure, one died from head and neck cancer, one died from acute respiratory failure, one died from lung cancer, and two from unknown causes. Forty-eight patients (65.8 %) had no evidence of treatment failure, and 25 patients (34.2 %) presented some type (local-regional-metastatic or mixed) of failure. Locoregional control failure rate (LRCFR): 18 patients (24.7 %) had local or loco-regional failures, and nine patients (12.3 %) had exclusive local failure; unfortunately, there were no data to precise if local failure occurred in the tumor bed or a distance. In regard to the regional control failure rate (RCFR), nine patients (12.3 %) presented with regional or loco-regional failure, and seven patients (9.6 %) presented with exclusive regional (node) failure. Two patients (2.7 %) presented with distant metastasis.

### Amputation surgery

Twenty patients (27.4 %) had amputation surgery, five (6.8 %) had total amputation surgery because of necrosis, and 15 (20.5 %) surgeries were performed because of failure, with five total penile amputation surgeries and 10 partial penile amputation surgeries (unknown for the others).

**Fig. 2:**
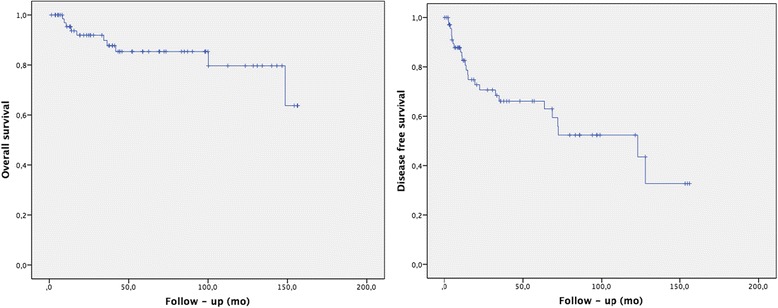
Overall survival and disease free survival

The median overall survival (OS) was 132 months, and OS rates were 95.3 % at 12 months, 85.4 % at 5 y and 79.7 % at 10 years (Fig. 2). The mean disease-free survival (DFS) was 90 months (2.6–127.9); DFS at 1, 5 and 10 years was 86.2, 66.3 and 52.5 %, respectively (Fig. 2). The 1-, 5- and 10-year DFS for local controls were 98.4, 88 and 84 %. The 1-, 5- and 10-year amputation-free survival was 92.7, 69.1 and 60.0 %, respectively. The 1-, 5- and 10-year regional control-free survival (RCFS) was 91, 87.3 and 83.5 %, respectively. The 1-, 5- and 10-year metastasis-free survival (MFS) was 100, 95.9 and 95.9 %. The median overall specific survival (OSS) was not discernable, but the mean OSS was 150 months, and the 1-, 5- and 10-year rates were 100, 95 and 95 %, respectively.

### Univariate and multivariate analysis

**Fig. 3:**
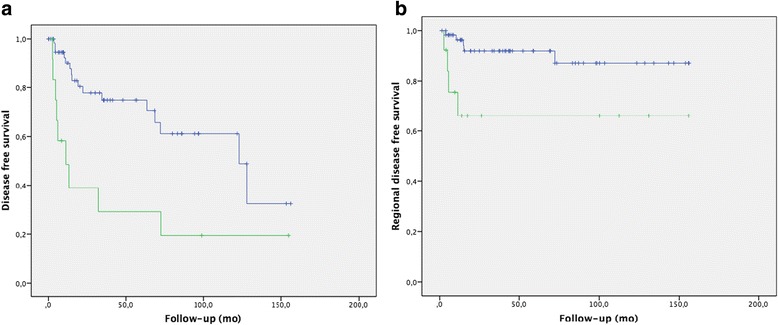
**a**) Disease free survival depending on nodal (*blue line* for patients node positive disease and *green* for patients node negative disease). **b**) Regional disease free survival, *blue line* for patients without node disease and *green* one for node negative disease

Node-positive disease at the time of diagnosis was associated with a relatively poor DFS, with a median of 11.3 months (IC95 % 0.8–21.7) versus 123 months (IC95 % (74.01–173.1) for node-negative disease (*p* = 0.002) and an RC with a mean RCFS of 140.6 months (IC95 % 127.4–153.8) versus 105 months (IC95 % 64.5–146.3) (*p* = 0.000) (Fig. 3). Diabetes was associated with a worse amputation-free survival (*p* = 0.001), with a mean survival of 78.7 months (IC95% (32.6–124.9)) versus 113.7 months (IC95 % (94.5–132.9)) in non-diabetic patients and tumor necrosis with amputation-free survival of 20.1 months (IC95 % 0–40.8) (*p* = 0.000) versus 115.8 months (IC95 % 98.1–133.4). Tobacco use was associated with a worse OS, with a mean OS of 110.8 months (IC95 % (82.46–139.2)) versus 145.6 (IC95 % 133.9–157.4) (*p* = 0.015) for non-tobacco users and tumor necrosis with a mean OS of 63.9 months (IC95 % 18,9–109) versus 139.6 months (IC95 % 127.8–151.4) (*p* = 0.001). No factor was statistically significant in multivariate analysis.

## Discussion

Penis cancer is a rare disease, and no international guidelines exist for making daily clinical decisions; the majority of reports are from single-center retrospective studies. Herein, we report our study conducted in a series of 73 patients with local and locally advanced squamous cell penile cancer treated with conservative intention by IBT. The median overall survival (OS) was 132 months, and the OS rates were 85.4 % at 5 years and 79.7 % at 10 years. The mean disease-free survival (DFS) was 90 months (range 2.6–127.9); 1-, 5- and 10-year DFS were 86.2, 66.3 and 52.5 %, respectively (Fig. 2). The 1-, 5- and 10-year rate of local control was 98.4, 88 and 84 %, respectively. The 1-, 5- and 10-year amputation-free survival was 92.7, 69.1 and 60.0 %, respectively The 1-, 5- and 10-year regional control-free survival (RCFS) was 91, 87.3 and 83.5 %, respectively.

Local control in early disease stages is equivalent when IBT and surgery are compared; even if local control is better in patients with all stages (T2 included) who have undergone operation than those who have been treated by brachytherapy after 5-years, conservative approaches must always be proposed to the patients because of the negative psychological impact on patients in the event of mutilation due to a penile procedure [10]. Although the presence of nodes plays an important role in the prognosis, local treatment is mandatory because no systemic treatment has stopped or inhibited the growth of the primary tumor in the penis.

Our local control (98.4, 88 and 84 % at 1, 5 and 10 years) and penis conservation rates (92.7, 69.1 and 60.0 % at 1, 5 and 10 years) are equivalent to those already described in the literature or even higher, as in this case of the study reported by Mazeron et al. [12]. In that study, 78 % of local control rates with penis conservation in 74 % of patients was reported for a series of 50 patients. Daly et al. [13] presented a series of 22 penile cancer patients treated by IBT, with only one local recurrence. Rozan et al. [14] presented a large series of 259 patients treated by IBT (184 patients) or surgery and IBT association (75 patients), with local control rates of 88 % at 3 years in both groups and penis conservation in 84 % of patients. Pimenta el al. [15] reported that only one in their series of 25 patients experienced early local failure (4 months after IBT) with a median follow-up time of 9.2 years. Crook et al. [16] reported a local control rate of 88 % at 48 months in their series of 67 patients. Soria et al. [17] reported 71.4 % of local control and penis conservation in their series of 35 penis cancer patients treated exclusively with IBT. Delannes et al. [18] presented local control rates with penile conservation in 67 % of all patients and 75 % of patients with T1-T2 disease with 23 % local necrosis (treated by local excision, partial amputation or total amputation) in a series of 51 patients. De Crevoisier [19] observed a 10-year penile cancer recurrence rate of 20 % in their series of 144 patients with invasive penis cancer treated exclusively with IBT. Cook et al. [20] reported a cumulative incidence of freedom from local failure of 85.3 % at 5 years. Delaunay et al. [21] reported that 60 % of the patients in their study had no recurrence after 80 months of follow-up.

In our series, the occurrence of late morbidity, mainly urethral stenosis and gland necrosis, was similar to that reported in historical data (Table 3). It is likely that the retrospective character of the study led to an underestimation of this measure, particularly in regard to urethral stenosis. The main factors associated with necrosis are the number of implanted wires, which is directly associated with tumor size and wire activity [14].

**Table 3:** Late morbidity

	De Crevoisier et al. [17]	Cook et al. [18]	Cook et al. [15]	Rozan et al. [13]	Pimenta et al. [14]	Mazeron et al. [11]	Soria et al. [16]	Delannes et al. [19]	Delaunay et al. [20]	Cordoba et al.
Urethral stenosis	18 %	12 %	9 %	30 %	43 %	16 %	1.3 %	45 %	21.1 %	6.8 %
Penis necrosis	4.8 %	16 %	12 %	21 %	1 %	6 %	1.3 %	23 %	–	6.8 %

Lymph node invasion is the most important prognostic factor in penis cancer. In this sense, a lymph node examination before curative local treatment is necessary, but clinical guidelines for this assessment do not exist. When there are no clinically detectable affected nodes, modified inguinal lymph node dissection (ILND) and dynamic sentinel-node biopsy (DSNB) can be performed; when there are clinically detected affected inguinal nodes, ILND must be performed. The retrospective character of our study does not allow for the evaluation of when ILND, modified ILND or DSNB were performed. It is suggested that adjuvant radiotherapy can be considered after complete ILND in patients with multiple or large inguinal lymph nodes or extra capsular extension [7]. Postoperative inguinal irradiation was performed independent of the number of positive nodes (one or more) and the status of the node capsule. Our results (1-, 5- and 10-year RCFS: 91, 87.3 and 83.5 %, respectively) confirm excellent regional control. Lymphedema has not been assessed in our series. No lymph node failure was observed when inguinal pN0 status was observed at diagnosis.

### Limitations

The main limitations of this analysis are the retrospective design and single-institution series.

In this regard, toxicity rates were likely under estimated, including associated sexual pain, trophic alterations after treatment, and urethral stenosis.

Pre-therapeutic nodal status was not evaluated uniformly (clinical examination, imaging, lymphadenectomy and sentinel node sampling were all applied for node evaluation).

IBT treatment can be performed using pulsed-dose-rate (PDR) [22] and high-dose-rate (HDR) [23, 24] brachytherapy techniques due to the cessation of commercialization of iridium wires.

The early local control and toxicity rates observed in our study are comparable to historical data using LDR techniques taking into account that median follow up is less than 5 years.

## Conclusions

IBT is an excellent treatment option for localized penis cancer in terms of local control and organ conservation. In our study, local control, amputation and toxicity rates were similar to those of previously published studies. Due to the rarity of this cancer, patients from different centers were pooled into groups to prospectively and adequately describe dose prescription and dose delivery, local control and toxicity rates, along with uniform daily clinical practice results analysis.

## Abbreviations

BT, brachytherapy; LDR, low dose rate; PDR, pulsed dose rate; CTV, clinical target volume; GTV, gross tumor volume; OS, overall survival; OSS, overall specific survival; PFS, progression-free survival; LFS, local disease-free survival; RFS, regional disease-free survival; LRFS, loco-regional disease-free survival; AFS, amputation-free survival; LRCFR, locoregional control failure rate; RCFR, regional control failure rate; ILND, inguinal lymph node dissection; DSNB, dynamic sentinel-node biopsy; HDR, high-dose-rate
