# Erratum to: Low-dose brachytherapy for early stage penile cancer: a 20-year single-institution study (73 patients)

**DOI:** 10.1186/s13014-016-0708-5

**Published:** 2016-09-28

**Authors:** Abel Cordoba, Alexandre Escande, Stephanie Lopez, Laurent Mortier, Xavier Mirabel, Bernard Coche-Déqueant, Eric Lartigau

**Affiliations:** Academic Radiation Oncology Department, Oscar Lambret Comprehensive Cancer Center, SIRIC ONCOLille and University Lille 2, 3 rue Fréderic Combemale, Lille, France

## Update and Erratum

The original version of this article [1] has been updated to include the full names of the authors. No other changes have been made to the author list other than the inclusion of the author’s first names.

## References

[1] Cordoba A (2016). Radiation Oncology.

